# Are brain metastases a special issue in POLE-mutated endometrial cancer? a case report series

**DOI:** 10.3389/fonc.2025.1568137

**Published:** 2025-09-01

**Authors:** Christoph Ebner, Kristina Lindemann, Gunnar Kristensen, Alice Frosch, Hanne Askautrud, Gianpaolo Di Santo, Katharina Leitner, Christian Marth, Alain G. Zeimet

**Affiliations:** ^1^ Department of Obstetrics and Gynecology, Medical University Innsbruck, Innsbruck, Austria; ^2^ Department of Surgical Oncology, Section for Gynecologic Oncology, Oslo University Hospital, The Norwegian Radium Hospital, Oslo, Norway; ^3^ Faculty of Medicine, Institute of Clinical Medicine, University of Oslo, Oslo, Norway; ^4^ Institute for Cancer Genetics and Informatics, Division of Cancer Medicine, Oslo University Hospital, Oslo, Norway; ^5^ INNPATH GmbH, Institute of Pathology, Innsbruck, Austria; ^6^ Department of Nuclear Medicine, Medical University Innsbruck, Innsbruck, Austria

**Keywords:** POLE mutation, brain metastasis, immunotherapy, endometrial cancer, molecular classification

## Abstract

POLE-mutated endometrial cancers have repeatedly shown to harbor an excellent prognosis. Recurrences or primary advanced disease are rare events in this subgroup. We report three cases of POLE-mutated endometrioid ECs with brain metastases. While patients with CNS metastases from endometrial cancers are very uncommon and generally confer a poor prognosis and limited survival, all cases presented here survived substantially longer than the median survival time of patients with brain metastasis published in the literature. All patients responded well to systemic therapy or radiotherapy. Additionally, we present one case with complete response to only four cycles of pembrolizumab monotherapy. These cases suggest that, even in the setting of brain metastases or recurrent disease, a pathogenic POLE mutation remains a marker for excellent clinical outcome. The question of why these tumors metastasized to the brain in the first place remains to be answered by further research.

## Introduction

Molecular classification of endometrial cancers (ECs) into four subgroups led to better risk stratification and was promptly integrated in treatment guidelines. Even in the 2023 renewed International Federation of Gynecology and Obstetrics (FIGO) staging system for EC molecular, properties like abnormal p53 expression or polymerase epsilon exonuclease domain mutation (*POLE*-EDM) are directly affecting tumor stage (1–3).

*POLE*-mutated (*POLE* ^mut^) ECs are usually high-grade, ultramutated cancers most commonly of endometrioid histology. These cancers come with an excellent prognosis, a fact that has been underscored in large cohorts and meta-analyses (4–6). The good prognosis of *POLE* ^mut^ tumors is maintained even in the presence of properties otherwise considered as high-risk such as substantial lymph vascular space invasion (LVSI), *TP53* mutation, high tumor grade, or aggressive histology (7, 8).Therefore, recent treatment recommendations insistently favor de-escalation of adjuvant treatment in early *POLE*-mutated EC irrespective of other additional risk factors. While the overall prognosis is excellent, most of the existing literature is focused on early stage disease and data of large cohorts of advanced *POLE* ^mut^ ECs is currently unavailable due to the rarity of these cases. One key factor to explain their excellent prognosis is thought to be caused by the high tumor mutational burden (TMB), resulting in the expression of abundant neoantigens, which make tumor cells highly visible for the host’s immune system (9).

Metastasis to the brain is a rare event in EC. Incidence is reported between 0.3% and 1.16%, but brain metastases are more common in advanced and recurrent cancers with an incidence of 3% and 6%, respectively. On the other hand, only 0.7%–0.84% of occurring brain metastases are estimated to originate from a primary cancer of the endometrium. The two ways of hematogenous metastatic spread to the brain are either via the vena cava, the heart, and the aorta or alternatively through the paravertebral (Baston) venous plexus (10–12).

The endometrioid histologic subtype is the most common subtype accounting for 53.3% of brain metastases derived from ECs. Among endometrioid carcinomas that metastasize to the brain, approximately 80% are of high-grade histology. Non-endometrioid subtypes appear disproportionately represented, given that they account for the remaining 47.7% of brain metastases, despite constituting only about 20% of ECs overall (13, 14). Furthermore, 70% of affected patients have additional extracranial metastases, most commonly in the lung, bone or liver. Data on the frequency of brain metastasis in the now routinely recognized molecular subgroups of EC is currently still missing.

## Case 1

A 31-year-old patient presented to the neurologic emergency room of the University Hospital Innsbruck with headache and lower extremity weakness. MRI imaging showed several cystic lesions in the right hemisphere of the brain. Whole body FDG PET CT detected a hypermetabolic area in the uterine corpus.

**Figure 1:**
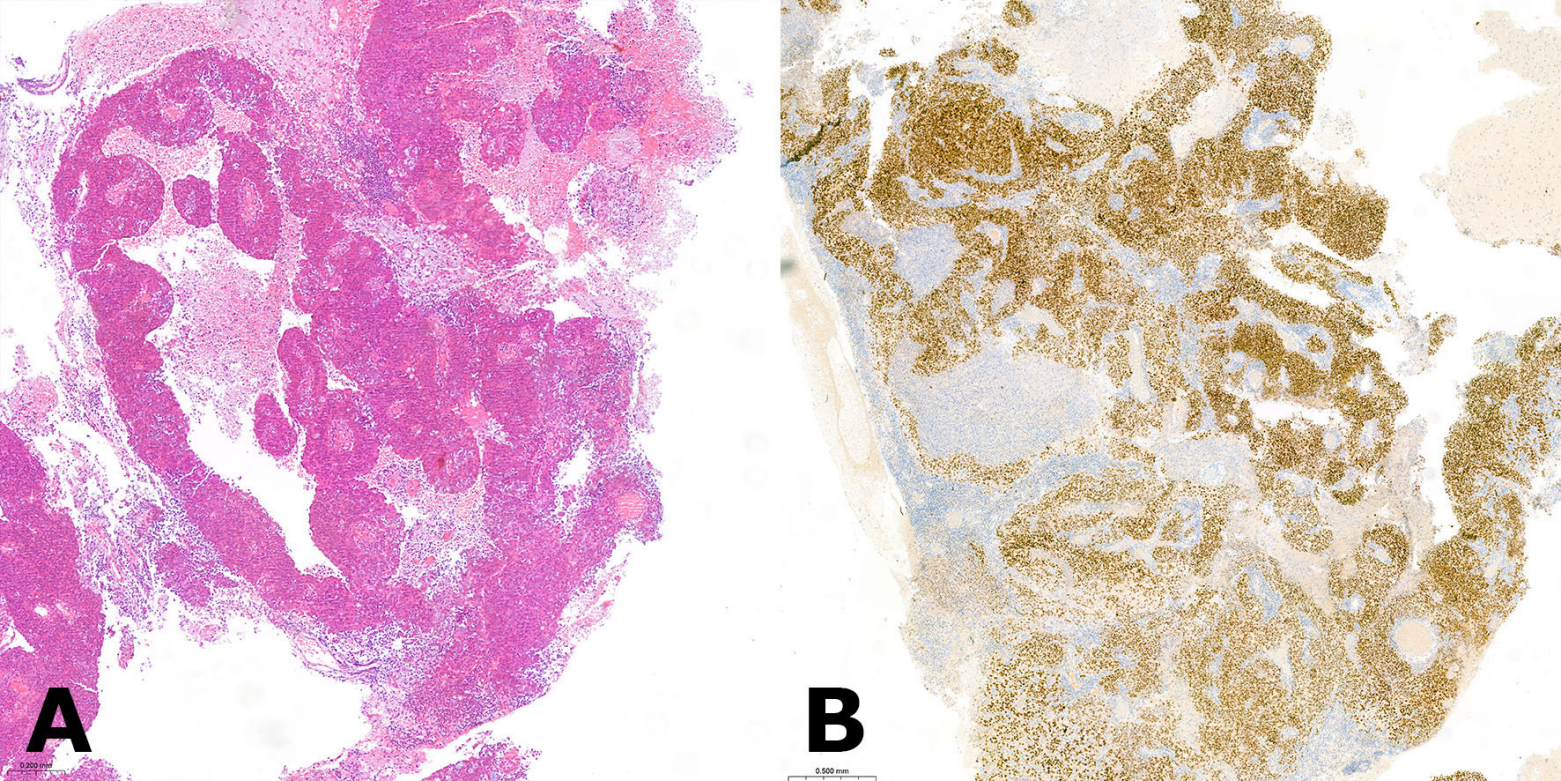
**(A)** Hematoxylin and eosin (H&E) stained section of the resected brain metastasis depicting a high-grade endometrioid with clustering of tumor cells around a central vessel, which is a typical feature of brain metastatic lesions. **(B)** The positive immunohistochemical stain for paired-box gene 8 (PAX8) was indicative of a gynecologic primary tumor of Mullerian origin.

The brain lesions were resected, and histologic workup showed a poorly differentiated carcinoma. Positive immunohistochemical (IHC) staining for PAX 8 hinted to a gynecologic primary (**Figure 1**). Next generation sequencing was performed and revealed a pathogenic *POLE*-EDM (p.A456P) and multiple pathologic mutations in other known cancer associated genes.

The patient was referred to the Department of Gynecology and Obstetrics. Transvaginal ultrasound showed adenomyosis like changes in the dorsal part of the uterine corpus with increased vascularity detected by doppler ultrasound. Dilation and curettage resulted in a histologic diagnosis of a poorly differentiated high-grade endometrioid EC. Standard IHC stains for endometrial cancer found no abnormal expression of p53 or mismatch repair (MMR) proteins. L1CAM status was negative. The uterine cancer specimen was also sequenced using NGS and mutations in *PIK3CA*, *PTEN* and *POLE*-EDM which were identical to those from the brain lesion were identified (**Table 1**).

**Table 1:** Molecular characteristics and treatment of the primary cancer and the brain metastases in Case 1.

	Uterine cancer	Brain metastases
*Next Generation Sequencing* selection of detected mutations	***POLE (p.A456P)*** PTEN (p.E299*) PTEN (p.Y174*) ***PTEN (p.F341C, VUS)*** ***PIK3CA (p.T1025A)*** *PIK3CA (p.N331H, VUS)* BRACA2 (pE1120*) ^†^ ***BRCA2 (p.R2896C, VUS)*** ***BRCA2 (p.F1698C, VUS)*** ***BRCA2 (p.K722N, VUS)***	***POLE (p.A456P)*** PTEN (p.E299*) PTEN (p.Y174*) ***PTEN (p.F341C, VUS)*** ***PIK3CA (p.T1025A)*** ***BRCA2 (p.R2896C, VUS)*** ***BRCA2 (p.F1698C, VUS)*** ***BRCA2 (p.K722N, VUS)***
*Immunohistochemistry*	ER positive PR positive PAX8 positive WT1 negative	ER positive PR positive PAX8 positive WT1 negative
*Tumor mutational burden*	high, 280 muts/Mb	high, 218 muts/Mb
*Microsatellite Stability*	MSS	MSS
*Treatment*	Immunotherapy with pembrolizumab followed by simple hysterectomie	Resection followed by whole brain radiation therapy and immunotherapy

ER, estrogen receptor; PR, progesterone receptor; muts/mb, mutations per megabase; MSS, microsatellite stable; VUS, variant of uncertain significance; ^†^This is a known pathogenic mutation, however it was only detected with an allele frequency of 3% in a specimen with 60% tumor content. * denotes a stop codon (nonsense mutation). Bold mutations are present in both the uterine cancer and the brain metastasis.

These findings proved that the endometrium was the primary site of this metastatic disease, leading to the final diagnosis of a *POLE* ^mut^ high-grade endometrioid EC in FIGO (2009) Stage IVB. This constellation of findings was unexpected as *POLE* mutation is generally associated excellent prognostic outcome and is rarely found in advanced disease.

The brain metastases as well as the primary tumor were sequenced again using the TrueSight oncology 500 gene panel. Many variants of unknown significance were detected and both tumor sites exhibit a very high tumor mutational burden of 280 muts/Mb (primary tumor) and 218 muts/Mb (brain metastases).

The patient underwent whole brain radiation and on the first checkup 3 months after diagnosis no signs of disease progression were found in the uterus, the brain or elsewhere. Due to the ultra-mutated phenotype, our patient received single agent immunotherapy with pembrolizumab after conclusion of radiation therapy. Checkup PET-CT following the fourth cycle of pembrolizumab showed no sign of a residual tumor in the uterus.

**Figure 2:**
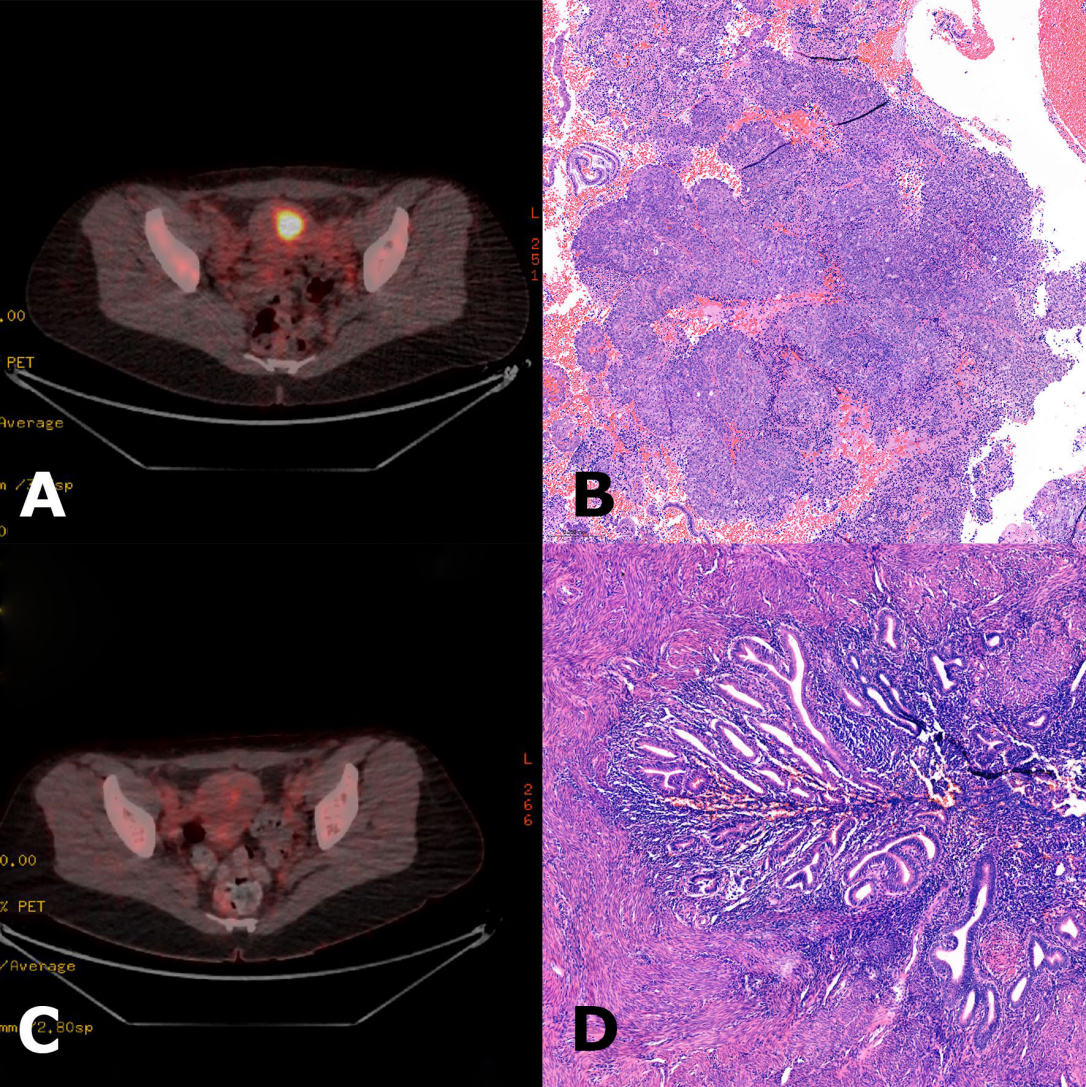
**(A)** Initial 18F-fluorodeoxyglucose positron emission tomography-computed tomography (FDG-PET-CT) scan showing high metabolic activity in the uterine corpus, suspicious for endometrial carcinoma. **(B)** Hematoxylin and eosin (H&E) stain of curettage material showing a high-grade endometrioid endometrial carcinoma. **(C)** FDG-PET-CT scan at follow-up after four cycles of pembrolizumab revealed a complete metabolic response. Simple hysterectomy was performed shortly after the PET scan. **(D)** H&E stain of the hysterectomy specimen showing predominantly regularly differentiated endometrium without signs of residual carcinoma. PET-CT images courtesy of the Department of Nuclear Medicine, Medical University Innsbruck.

A total laparoscopic hysterectomy was performed without complications and histologic examination revealed only complex endometrial hyperplasia but no residual invasive carcinoma (**Figure 2**). Immunotherapy with pembrolizumab was continued after surgery. The therapy was very well tolerated, and the patient is now 14 months after primary diagnosis in followed-up without evidence of progression.

## Additional cases

Two additional patients with brain metastases from POLE^mut^ EC were identified in a retrospective cohort study of patients who underwent treatment at the Norwegian Radium Hospital, Oslo University Hospital. The cohort is described elsewhere. Retrospective molecular classification was performed on archived tissue. Allele-specific PCR for the five hotspot *POLE*-EDMs (P286R, V411L, S297F, A456P, S459F) was performed instead of *POLE* sequencing. IHC was performed to detect aberrations in MMR protein and p53 expression (5).

## Case 2

This patient initially presented in 2016 with FIGO (2009) stage IA endometrioid EC grade 2 without LVSI and was treated with surgery. Retrospective molecular analysis of this tumor revealed a pathologic *POLE*-EDM (p.V411L) as well as a loss in MMR protein PMS2 and according to the FIGO 2023 classification would now be categorized as stage IAm_POLEmut_. IHC showed regular expression of p53, L1CAM negativity and estrogen receptor positivity. Two years later this patient presented with metastatic disease to the brain and the lung. Stereotactic radiation of brain metastases was performed, and the patient underwent four cycles of chemotherapy with carboplatin and paclitaxel, leading to a complete response followed by a consolidation therapy with exemestane. After 3.5 years another relapse in the lungs was diagnosed and was successfully treated with stereotactic radiation and treatment with progestins. One year after, the patient experienced a third episode of recurrent disease manifesting again as metastases to the brain and the lung. Despite abstaining from further treatment, the patient is still alive.

## Case 3

This patient was initially treated with surgery for an endometrioid EC grade 3 FIGO (2009) stage IB with LVSI. In the retrospective molecular analysis a *POLE*-EDM (p.P286R) was found. MMR proteins, p53 and estrogen receptors were regularly expressed, L1CAM was negative. Very shortly after the diagnosis brain metastases were detected with MRI and were surgically resected one month after the primary surgery. The patient underwent chemotherapy and unfortunately recurred with brain metastasis 6 months later. This cerebral metastasis was again resected, and adjuvant whole brain radiation was performed. A second relapse to the brain occurred 9 months later and was also treated with radiotherapy. The patient died 4.5 years after the primary diagnosis.

## Discussion

The presented cases represent ECs with two very distinct and rare characteristics namely a pathological *POLE*-EDM and the development of brain metastases in their course (**Table 2**). To our knowledge, association between these two events has not been comprehensively investigated so far.

**Table 2:** Clinical presentation, timeline of treatment and outcomes of the presented cases of *POLE* ^mut^ endometrioid carcinoma with brain metastases.

Case	Histology	Presentation	Treatment and outcome
1	endometrioid EC, G3 *POLE*-EDM (p.A456P) pMMR p53 wild-type ER positive	FIGO (2009) Stage IVB; detection of brain metastases due to neurological symptoms before uterine tumor was detected	resection of brain metastases followed by whole brain radiation and pembrolizumab monotherapy, simple hysterectomy after four cycles of pembrolizumab pCR; now pembrolizumab maintenance, no evidence of disease 14 months after diagnosis
2	endometrioid EC, G2 *POLE*-EDM (p.V411L) dMMR (PMS2 loss) p53 wild-type ER positive	FIGO (2009) Stage IA; recurrent disease with brain and lung metastases	initially surgery, multiple relapses to the brain and lung managed with radiation, chemotherapy and hormonal therapy; patient is still alive 8 years after diagnosis and 6 years after detection of brain metastases despite no further treatment was initiated after the last relapse
3	endometrioid EC, G3 *POLE*-EDM (p.P286R) pMMR p53 wild-type ER positive	FIGO (2009) IB, Brain metastases detected shortly after surgery	initially surgery and resection of the brain metastases followed by chemotherapy, repeated brain metastasis resection, whole brain radiation; patient died after 4.5 years

*POLE*-EDM, polymerase epsilon exonuclease domain mutation; dMMR, mismatch repair deficient; pMMR, mismatch repair proficient; ER, estrogen receptor.

Stasenko et al. reported two cases of patients with brain metastases from *POLE* ^mut^ endometroid EC. One Patient initially presented with FIGO (2009) Stage IB disease and had a recurrence with brain metastases 20 months after primary therapy. After a follow-up of 42 months this patient had no evidence of disease. The other patient presented with FIGO stage IV disease and subsequently progressed with brain metastases. This patient died after 33 months of initial diagnosis (4).

The excellent prognosis of *POLE* ^mut^ ECs has been shown repeatedly in large cohorts. Nevertheless, advanced, primary metastatic as well as local and distant recurrences have been reported in this molecular subtype. An important question regarding these cases is whether special properties of the cancer itself (e.g., specific *POLE* mutations, a particular genomic or epigenetic context) or patient-related attributes (e.g., host’s immune competence), are responsible for the unfavorable disease dissemination.

Generally, brain metastases either primary or in the recurrent setting are associated with an extremely poor prognosis in all cancers. Median survival after the diagnosis of brain metastasis from ECs is described to be 6.8 months (13). However, patients reported here survived substantially longer and responded very well to various treatment approaches. It seems that even in the setting of primary advanced tumor spread and recurrent disease the prognosis of *POLE^mut^* ECs appears to remain far superior to tumors of other molecular subgroups.

Although no reliable data on the incidence of brain metastases in *POLE* ^mut^ EC do exist, it remains speculative whether the brain is not representing a predestinated localization for tumor spread in *POLE* ^mut^ EC. In order to protect the brain against fatal immune mediated damages from destructive inflammation, the local brains’ immune response is thought to be significantly attenuated in comparison to peripheral tissue (15, 16). Therefore, *POLE* ^mut^ EC despite its high visibility to the host’s immune system by the large number of neoantigens presented could take advantage of the locally reduced immune surveillance in spreading to the brain. The influence of the molecular subtype of EC on the potential for CNS spread has yet to be investigated. Even in cancer types with known brain tropism, no universally applicable molecular risk profile has been found that is valid across various cancers. Breast and lung cancers that metastasize to the brain were shown to utilize the cell adhesion molecule L1CAM for migration along capillaries (17). Although, in EC L1CAM overexpression is closely related to poor outcome as well in endometrioid as in serous subtypes no overexpression was revealed in the herein described EC showing CNS spread (18).

Generalization based on this report should be limited due to the small sample size (*n* = 3) and the lack of systematic evaluation in large cohorts. This is a serious risk for selection bias. Nonetheless, given the rarity of this metastatic pattern in *POLE* ^mut^ EC, our case series provides clinically relevant insights and raises questions for possible further research. A large multicenter evaluation or an international registry would be needed to comprehensively describe the dissemination pattern of this subgroup of EC and investigate whether the brain is a preferred region of metastases. In the context of this research, potential confounding molecular alterations occurring together with pathogenic *POLE* mutations explaining brain tropism could be identified.

## Conclusion

Despite efforts to de-escalate adjuvant treatment at least for early *POLE* ^mut^ EC as recommended in the recent guidelines, it remains open how these reported uncommon courses from at least two early stage cases will influence the discussion about under- and over-treatment of *POLE* ^mut^ EC. However, currently available data is insufficient to abandon general de-escalating strategies (2, 19). Relative to comparable cases of EC from other molecular subtypes, our report clearly shows that *POLE* ^mut^ EC with brain metastases nonetheless appear to exhibit better treatment responses and are obviously related to a more favorable clinical outcome. The identification of reliable factors able to predict such unusual systemic dissemination in *POLE* ^mut^ EC should be an urgent goal of the future research on this molecular subtype.

## Data Availability

The original contributions presented in the study are included in the article/supplementary material. Further inquiries can be directed to the corresponding author.
